# Immune evasion impacts the landscape of driver genes during cancer evolution

**DOI:** 10.1186/s13059-024-03302-x

**Published:** 2024-06-26

**Authors:** Lucie Gourmet, Andrea Sottoriva, Simon Walker-Samuel, Maria Secrier, Luis Zapata

**Affiliations:** 1 Department of Genetics, Evolution and Environment, UCL Genetics Institute, University College London, London, UK; 2 UCL Centre for Computational Medicine, University College London, London, UK; 3 Centre for Evolution and Cancer, Institute of Cancer Research, London, UK; 4 Computational Biology Research Centre, Human Technopole, Milan, Italy

**Keywords:** Cancer Hallmarks, Cancer Evolution, Immunogenomics, immune evasion, natural selection

## Abstract

**Background:**

Carcinogenesis is driven by interactions between genetic mutations and the local tumor microenvironment. Recent research has identified hundreds of cancer driver genes; however, these studies often include a mixture of different molecular subtypes and ecological niches and ignore the impact of the immune system.

**Results:**

In this study, we compare the landscape of driver genes in tumors that escaped the immune system (escape +) versus those that did not (escape −). We analyze 9896 primary tumors from The Cancer Genome Atlas using the ratio of non-synonymous to synonymous mutations (dN/dS) and find 85 driver genes, including 27 and 16 novel genes, in escape − and escape + tumors, respectively. The dN/dS of driver genes in immune escaped tumors is significantly lower and closer to neutrality than in non-escaped tumors, suggesting selection buffering in driver genes fueled by immune escape. Additionally, we find that immune evasion leads to more mutated sites, a diverse array of mutational signatures and is linked to tumor prognosis.

**Conclusions:**

Our findings highlight the need for improved patient stratification to identify new therapeutic targets for cancer treatment.

**Supplementary Information:**

The online version contains supplementary material available at 10.1186/s13059-024-03302-x.

## Background

Cancer is a highly prevalent disease defined by genetic instability and the accumulation of mutations. In the 1970s, Peter Nowell described cancer as a multistage process subject to different selective pressures [1]. Somatic mutations in driver genes promote cancer by providing cells with a proliferative advantage. Over the last decade, many studies have focused on identifying these positively selected driver genes [2–5]. However, the driver gene landscape is not fully understood as more genes are being identified through increased sequencing power. In addition to driver mutations, passenger events, with no impact on tumorigenesis, occur randomly and provide a trace record left by mutagenic exposures [6, 7]. Meanwhile, negative selection eliminates cells carrying deleterious or antigenic mutations [8–11]. To detect genes under selection in cancer, the ratio of nonsynonymous to synonymous mutations, or dN/dS, is commonly used [3, 5, 12, 13]. Genes under positive selection harbor more protein-altering mutations than expected by chance (dN/dS > 1), unlike genes under negative selection that are depleted of such mutations (dN/dS < 1). The mutational background is important as mutational processes can strongly influence the frequency of trinucleotide changes observed in the genome and can affect selection metrics [14].

Cancer immunoediting is an evolutionary process in which cancer clones undergo genetic changes under selection for low immunogenicity or the ability to escape immune recognition [15, 16]. These changes can include the accumulation of mutations that create new peptides called neoantigens, which can trigger an immune response. When a neoantigen is processed by the major histocompatibility complex (MHC) and displayed on the tumor surface, neoantigen carrying cells can be eliminated by cytotoxic lymphocyte T cells or natural killer cells [17]. To avoid cell death, cancer cells can develop mechanisms, such as depletion of the neoantigen pool [18], impairment of antigen presentation [18], downregulation of tumor antigen expression [19], or overexpression of immune checkpoint proteins [20]. A recent study [21] has shown that active immune surveillance can lead to fitness trade-offs between oncogenic and immunogenic mutations, shaping the distribution of mutations in cancer genomes. Based on these findings, we hypothesize that classifying tumors based on their capacity of adaptive immunity (non-escaped or escape − versus escaped or escape +) will reveal novel driver genes and unmask differential mutational processes.

Here, we investigated the differences in genetic drivers between tumors that have evaded the immune system (escape +) and those that have not (escape −). We classified patients into two groups based on mutations in immune-related genes and compared the selective pressures on these genes as measured by dN/dS. We identified 85 driver genes that were linked to the presence or absence of an active immune response. We also found that some known cancer-causing genes had mutations in specific hotspots only in the escape − group, suggesting that the immune system plays a significant role in shaping the distribution and frequency of genetic drivers in tumors. Additionally, we found that patients with tumors that had escaped the immune system had worse overall survival, possibly due to the lack of immune control against genetic changes associated to neoantigens.

## Results

### Immune escape leads to neutral-like evolutionary dynamics measured by dN/dS

**Fig. 1:**
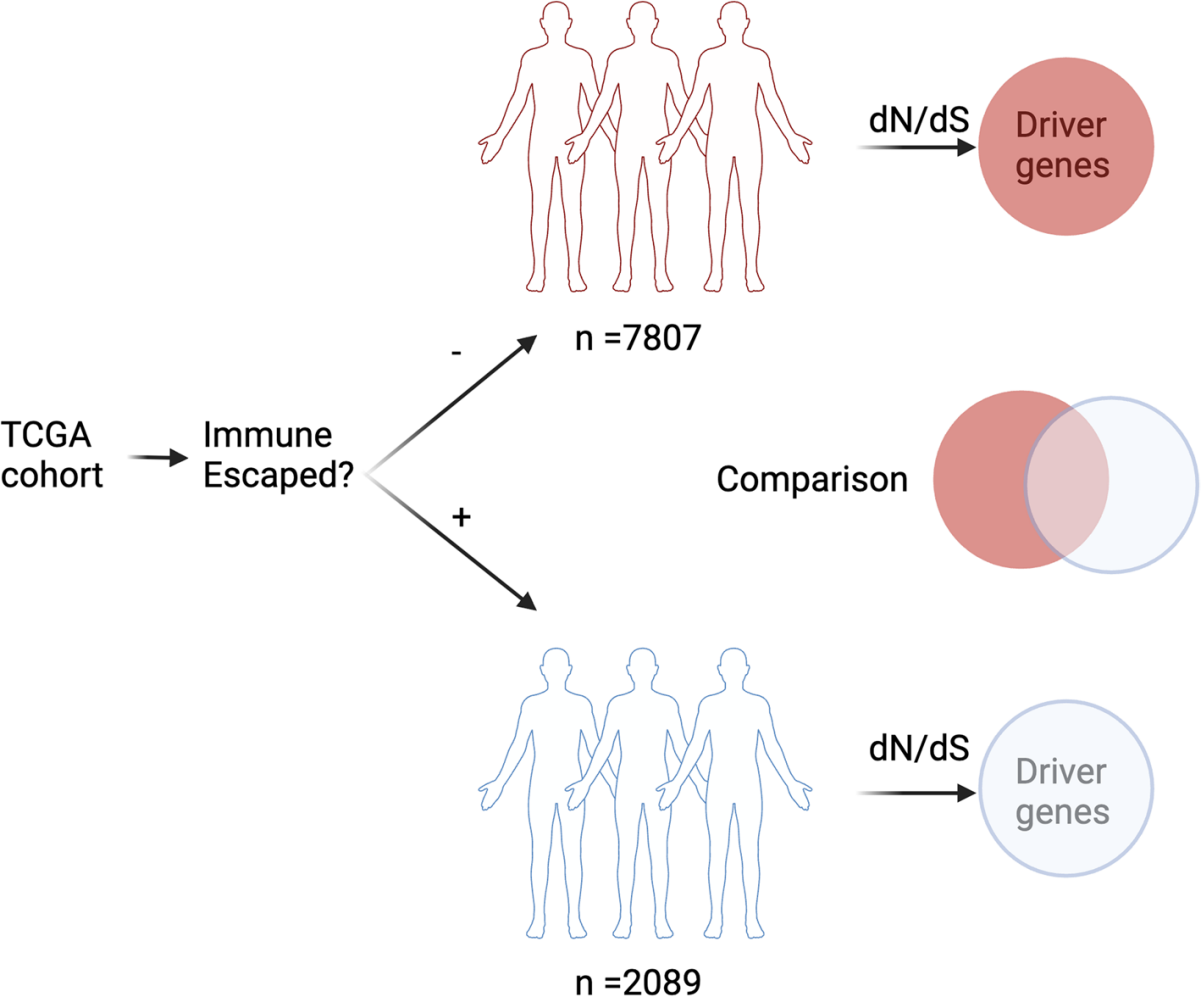
9896 tumors across 31 cancer subtypes from TCGA were classified into escape + and escape − based on mutations presented in the antigen presenting machinery. These two cohorts were then analyzed to detect genes under significant selection using dN/dS. Genes under significant selection can be used as molecular targets to improve cancer treatment strategies

To determine the impact of immune evasion in the selective landscape of tumorigenesis, we obtained a catalog of 88 genes involved in the antigen presenting machinery or previously associated to immune evasion [18] (defined as “escape genes,” Additional file 2: Table S1). We classified 9896 TCGA patients from 31 different cancer subtypes into escaped (escape +) and non-escaped (escape −) cohorts based on the presence of a non-silent point mutation in one of these genes (Fig. 1). These resulted into 2089 escape + individuals with an average tumor mutation burden per patient (TMB) of 426—over 4 times higher than the average TMB for the 7087 escape − patients (95 mutations per individual, Additional file 2: Table S2). Specifically, we observed that the average number of mutations per individual in escape + was 4.51 times higher for missense and 3.95 times higher for truncating mutations compared to escape − tumors. Other mutation types, such as essential splice sites, missense and nonsense events, were also higher in escape + compared to escape − (Additional file 1: Fig. S1). When considering at least a single mutation in one of these genes, there was a heterogeneous proportion of escape patients between tumor types, i.e., TCGT had the lowest proportion of only 1% escape + patients versus SKCM that had 51% of escape + patients (Additional file 2: Table S2, and Additional file 1: Fig. S2). When performing hierarchical clustering of escape gene frequencies (Additional file 2: Table S3), we observed tumors with a similar profile of immune evasion, i.e., lung and melanoma tumors (Additional file 1: Fig. S3).

**Fig. 2:**
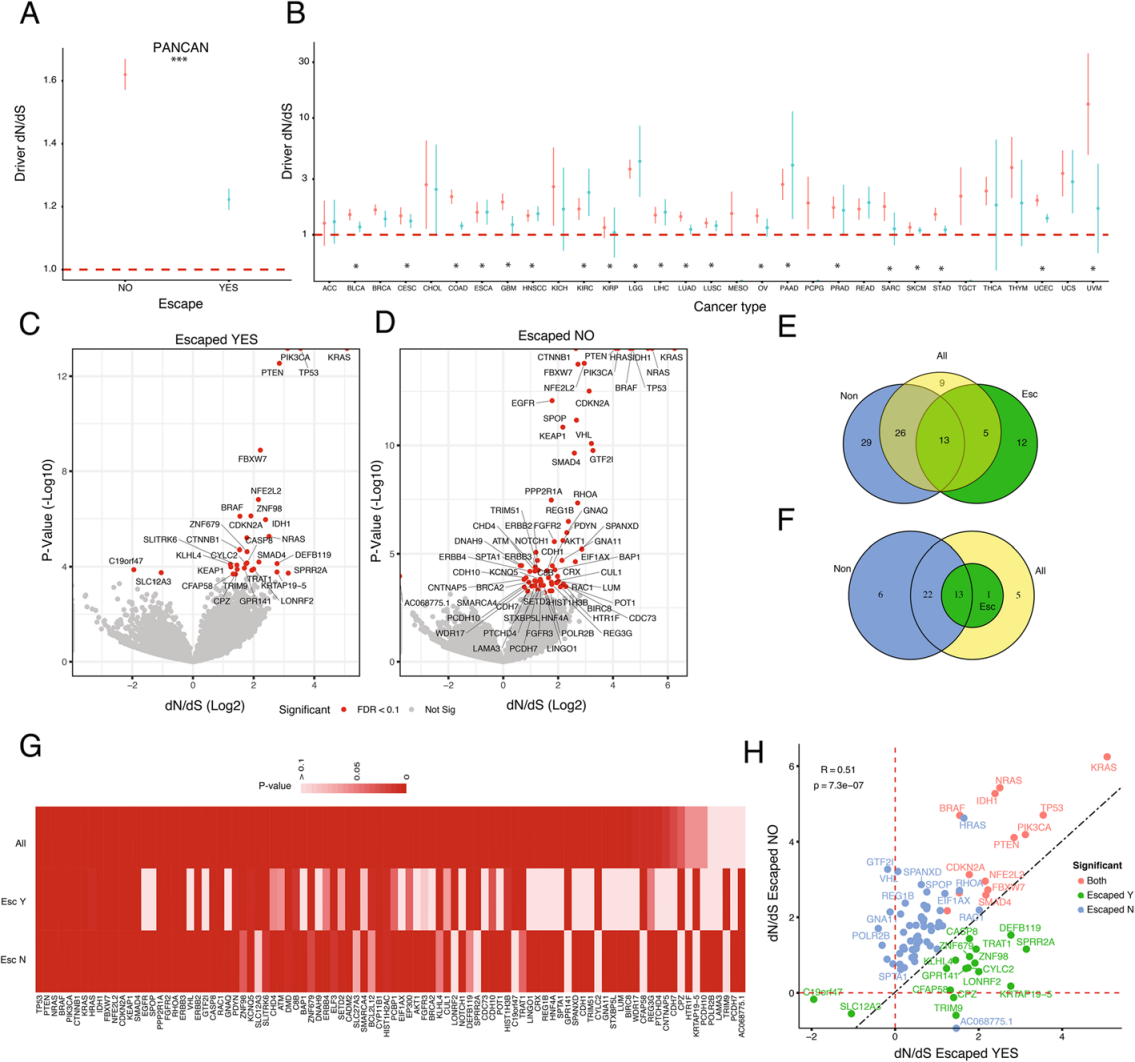
Selective landscape in escape + and escape − tumors. **A** Overall driver dN/dS for escape + and escape − tumors across all TCGA tumor types. **B** Overall driver dN/dS for 31 cancer subtypes separated by escape status (red—escaped − , blue—escaped +). Volcano plot for gene-level dN/dS using missense mutations versus *p*-value (Log10). Venn diagram showing the number of significant driver genes (*Q*-value < 0.1) using missense mutations considering **E** all genes or **F** restricted to known driver genes when separating by escape group or with all samples together. **G** List of significant driver genes using missense mutations in at least one group. **H** dN/dS value for significant genes in escaped − versus escaped + groups

We then calculated dN/dS on missense and truncating mutations for the escape + and escape − cohorts. We first calculated cohort *global dN/dS*, using all 19,562 protein coding genes, and *driver dN/dS*, using 366 known driver genes. At the pancancer level, global dN/dS (Additional file 1: Fig. S4, Escape + : 1.05, CI = [1.041:1.051], Escape − :1.07, CI = [1.060:1.072], Wilcoxon-Mann *p*-value = 4.45e − 6), and driver dN/dS (Fig. 2A, Escape + :1.223, CI = [1.189:1.257], Escape − :1.619, CI = [1.57:1.669], Wilcoxon-Mann *p*-value = 0.0067) were significantly lower and closer to neutrality in escape + compared to escape − , suggesting different evolutionary trajectories for each group. To control for possible mutation burden bias, we randomized the list of 88 “escape genes,” exclude patients classified as escape + , and calculate driver dN/dS. We found that driver dN/dS of the “random escape + ” was significantly higher than the driver dN/dS of the true escape + group (Additional file 1: Fig. S5, Random escape + dN/dS ~ 1.5 vs True escape + dN/dS ~ 1.22).

When looking at each tumor type, global dN/dS was significantly higher than one in 25 out of 31 cancer types in escape − patients compared to 19 out of 31 tumor types in escape + patients (Additional file 1: Fig. S6). Driver dN/dS was significantly higher than one in 29/31 escape − tumors compared to 20/28 escape + tumors. Overall, global dN/dS was similar between escape − and escape + , with a majority (21 out of 31) of escape − having higher global dN/dS compared to escape + . In ACC, GBM, and UVM, global dN/dS was significantly higher in the escape − group, whereas in KIRP, global dN/dS was significantly higher in the escape + group. Driver dN/dS of escape − was higher in several cancer types (BLCA, COAD, ESCA, GBM, LIHC, LUAD, LUSC, STAD, UCEC) compared to escape + (Fig. 2B). Moreover, when including deletions and point mutations in “escape genes” to classify escape status, driver dN/dS for escape + was not significantly different from one (Additional file 1: Fig. S7). Similarly, when including overexpression of PDL1 (Additional file 1: Fig. S8), as an orthogonal evasion mechanism, driver dN/dS was closer to one for escape + compared to escape − .

Next, we hypothesized that if global and driver dN/dS are different between escape + and escape − groups, the driver gene landscape would also be different. dN/dS analysis using missense and truncating mutations in all genes revealed 85 significant driver genes across pancancer (Additional file 2: Table S2). For missense mutations, there were 30 and 68 significant genes in escape + (Fig. 2C) and escape − (Fig. 2D) tumors, respectively. Seventeen out of 30 were escape + specific and 55 out of 68 were escape − , with 13 driver genes common to both groups. For truncating events, we found 64 and 41 driver genes for escape − and escape + tumors, respectively, with 33 out of 64 escape − , 10 out of 41 escape + , and 31 common to both (Additional file 1: Fig. S9). To determine whether stratifying patients into molecular subgroups revealed novel driver genes, we calculated dN/dS using all patients together. Twenty-nine out of 68 significant genes in escape − were missed when mixing patients (Fig. 2E). Similarly, 12 driver genes were only found in the escape + group, highlighting the impact of mixing patients with different evolutionary paths into cohort analysis for cancer driver discovery. Moreover, if we restrict this analysis to only known driver genes, we still observed 6 genes under significant selection only in the escape − group, which were missed when combined with escape + patients (Fig. 2F). Importantly, when combining all patients to predict driver genes, the majority still has a significant *p*-value (89 out of 94 genes) but lost significance after multiple testing correction (Fig. 2G), hence the importance of properly stratifying groups.

Among 55 escape − specific genes, the majority (41/55) were evolving neutrally in the escape + group despite having a similar number of mutations (Additional file 1: Fig. S10). For escape + specific significant genes, 15/17 have small signals of positive selection in the escape − group with values closer to neutrality (Additional file 1: Fig. S11). Interestingly, two genes were under significant negative selection in the escape + group: SLC12A3 and C19orf47. Among the common genes, there was a significant higher dN/dS in 9 out of 13 genes in the escape − compared to escape + , and all were previously known drivers (Additional file 1: Fig. S12). Driver dN/dS of escape − versus escape + was significantly correlated in the pancancer analysis (Fig. 2H) and in most cancer types (Additional file 2: Table S4). Few genes acted as strong drivers in one group while being completely neutral in the other. In the escape − group, these drivers included GTF2I, VHL, REG1B, SPANXD. In the escape + group, these included CPZ, CRTAP19-5, and CFAP58. GTF2I and REG1B are associated with negative regulation of angiogenesis and antimicrobial humoral immune response. VHL is involved in cell morphogenesis and the negative regulation of apoptotic process. Allele frequencies of driver genes in pancancer (Additional file 1: Fig. S13) and per-cancer (Additional file 1: Fig. S14) were significantly higher in driver compared to escape genes, suggesting that early clonal expansions precede acquisition of evasion mechanisms.

### Mutations are evenly distributed across driver genes in immune-escaped patients

**Fig. 3:**
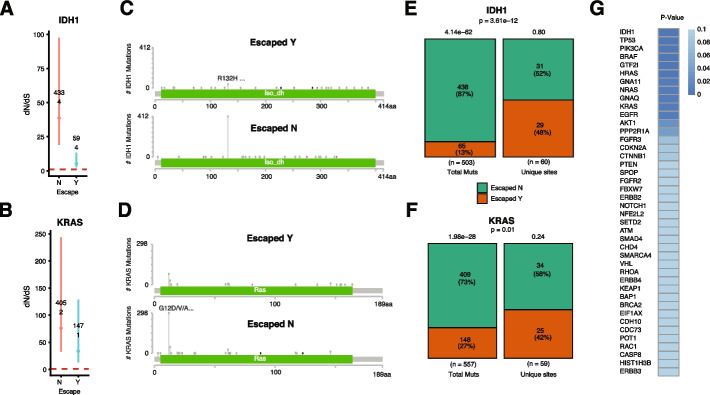
Non-random mutational distribution in non-escaped tumors. dN/dS for **A** IDH1 and **B** KRAS in escape − (red) and escape + (blue) groups including number of nonsynonymous (upper number) and synonymous (lower number) mutations. Lolliplots for mutations in escape − and escape + tumors for **C** IDH1 and **D** KRAS. Chi-square test comparing mutation number and unique mutated sites for **E** IDH1 and **F** KRAS. **G** Chi-2 *p*-value for significant known driver genes comparing the number of mutations versus the number of sites in both groups

To determine the immune system’s impact on the driver genes landscape, we explored whether mutations occurred more often at specific driver sites in escape + and escape − groups. We first compared two significant driver genes, IDH1 and KRAS. While IDH1 dN/dS was significantly higher in the escape − group (dN/dS ~ 31 versus dN/dS ~ 3, Fig. 3A), KRAS dN/dS was higher but not significantly different (dN/dS of 75 versus dN/dS of 35, Fig. 3B) compared to escape + . Known hotspots for IDH1 (Fig. 3C, position R132) and KRAS (Fig. 3D, position G12) were the most frequently mutated in both groups. Somatic mutations were more abundant in the hotspot of escape − compared to escape + group, while the number of unique sites mutated remained similar (Fig. 3E, IDH1: chi-square *p*-val = 3.6e − 12, Fig. 3F KRAS: chi-square *p*-val = 0.01). We then performed the same test on 55 significant “de novo” driver genes together and found the same pattern of mutations occurring preferentially at specific sites (i.e., hotspots) in escape − patients while occurring more evenly distributed across the gene in escape + patients (Fig. 3G, Pandriver *p*-value = 2.49e − 53). This was the case for known driver genes such as BRAF (*p*-value = 3.01E − 08), TP53 (*p*-value = 3.41E − 11), EGFR (*p*-value = 0.00877), and GNA11 (*p*-value = 0.00689).

To test whether the greater number of escape − individuals was a confounding factor, we repeated our analysis by downsampling the number of escape − patients to match the number of escape + patients. We found that fewer unique sites were mutated in the escape − group in IDH1, but not in KRAS, with respect to all mutations (Additional file 1: Fig. S15). This result suggests that there is a trade-off, at least for some driver genes, between oncogenicity and immunogenicity of the mutations accumulated in certain mutational hotspots. We then removed mutations in the most common hotspots of IDH1 and KRAS (R132 and G12, respectively). We found that for IDH1, the difference was not significant, suggesting that it is the only non-immunogenic hotspot, while for KRAS, there was still a significant difference, possibly associated to multiple non-immunogenomic hotspots (Additional file 1: Fig. S16). To determine whether oncogenes and tumor suppressors would be equally affected by escape status, we compared the proportion of mutations and found that oncogenes have more mutations in escape − patients compared to escape + (Additional file 1: Fig. S17). Interestingly, we also looked for selective differences between escape + and escape − in specific molecular subtypes. We found differences in ER + but not in ER- breast cancer patients (Additional file 1: Fig. S18), a reverse signal in HPV + compared to HPV − head and neck cancer patients (Additional file 1: Fig. S19), and overall minimal differences in HBV or HCV positive and negative patients of liver carcinoma (Additional file 1: Fig. S20).

### Mutational signatures associated to immune evasion in cancer

**Fig. 4:**
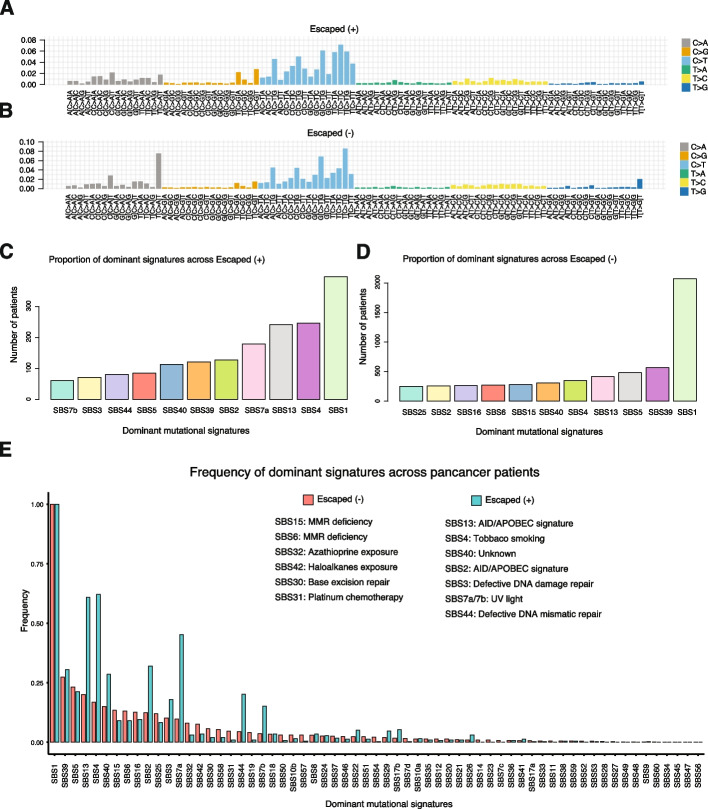
Mutational signatures associated to immune evasion. Proportion of mutation substitution in 96 trinucleotide contexts for **A** escaped + and **B** escaped − tumor cohorts. Dominant signatures per patient in **C** escaped + and **D** escaped − tumor cohorts. **E** Distribution of frequency of the top 60 signatures in escaped − tumors and their distribution in escaped + tumors

To determine the mutational signatures associated to immune evasion, we ran deconstructSigs [22] on both groups. We found a different profile in the trinucleotide substitutions between escaped + (Fig. 4A) and escaped − (Fig. 4B) tumors, especially in sites associated to C > A substitutions in a TCT context and C > T substitutions in a TCA or TCC context.

We tested whether immune escape allows a broader repertoire of mutational signatures to occur by comparing the most dominant signatures in escaped and non-escaped individuals. Although SBS1 signature was the most dominant in both groups, a greater proportion of escape + individuals (Fig. 4C) exhibited an alternative dominant signature compared to escape − (Fig. 4D). The top signatures for the escape + group were SBS4 (smoking), SBS13 (APOBEC), SBS7a (UV exposure), and SBS2 (APOBEC). In contrast, escape − signatures followed a flatter distribution with SBS39 (unknown) and SBS5 (clock-like unknown) being the second and third more frequent signatures. We next compared the top differential signatures between escaped + and escaped − . Interestingly, we found that immune evasion was associated to APOBEC, tobacco, and UV light signatures, while escape − tumors harbored signatures associated to mismatch repair deficiency and to various chemical exposures (Fig. 4E). We also investigated differences in mutational signatures between escape groups per cancer type (Additional file 1: Fig. S21) and found that 16 out of 31 tumor types have at least one mutational signature significantly different between groups. Escape + lung adenocarcinomas had a significant higher frequency of SBS4 (tobacco associated), compared to escape − tumors, suggesting that tobacco smoke leads to neoantigen accumulation accelerating the acquisition of immune evasion mechanisms. When controlling for mutation burden, we found that 46 out 49 mutational signatures tested display a significant difference between groups (Additional file 2: Table S5).

### Immune inflammation leads to better prognosis in the absence of immune escape

**Fig. 5:**
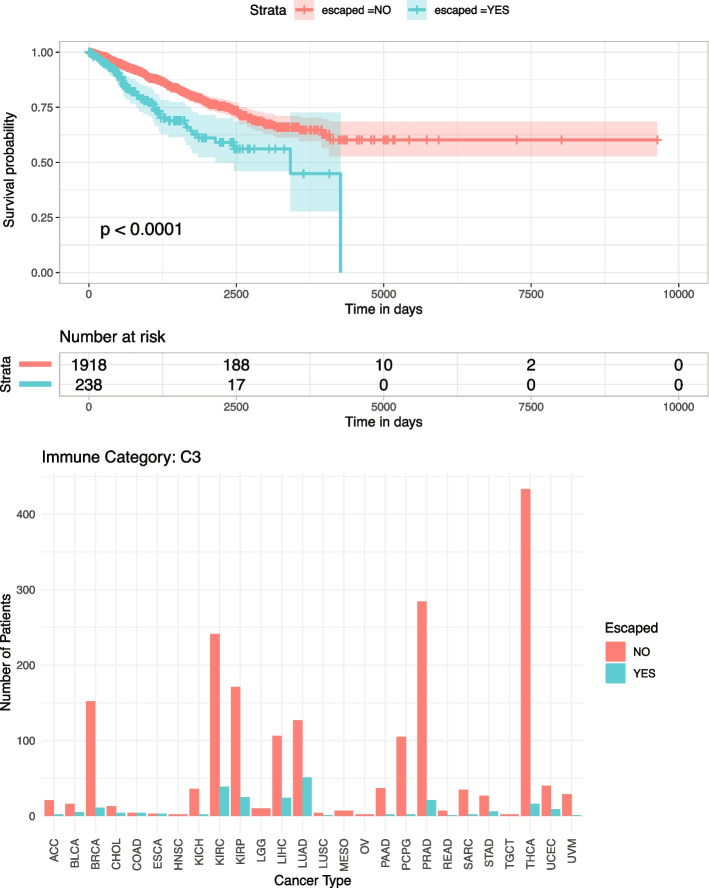
Survival and proportion comparison for escape + versus escape − patients classified as Inflammatory group (referred as C3) from Thorsson et al.

To determine whether classifying tumors into immune categories can reveal a difference on the clinical prognosis between immune escaped and non-escaped tumors, we classified patients into 6 categories previously defined by Thorsson et al. [23] (Additional file 2: Table S6). We found that the only pancancer category where there was a significant overall survival difference between escape − and escape + cohort was C3 (Fig. 5, *p*-value = 0.0001), which was characterized by an inflammatory signature. The other immune categories displayed no significant survival advantage (Additional file 1: Fig. S22), suggesting that the inflammatory response is a major factor responsible for long-term neoantigen-mediated immune surveillance. Immune-escaped tumors from the inflammatory cluster had a lower median overall survival time compared to non-immune escaped tumors possibly associated to the absence of immune control. An observation which was recently demonstrated in long-term survivors of pancreatic cancer [24], which have stronger immunoediting signals compared to short-term survivors, characterized by weak immunoediting and high intratumoral heterogeneity.

In our analysis of individual cancer types, thyroid carcinoma emerged as a prominent cancer within the inflammatory group (Fig. 5). Additionally, we found that escape + patients with bladder cancer (BLCA) and cervical squamous cell carcinoma (CESC) had improved survival rates. Conversely, escape − patients with mesothelioma (MESO), thyroid carcinoma (THCA), and thymoma (THYM) had a higher survival (Additional file 1: Fig. S23). These findings point to significant survival differences between escape + and escape − groups on a global scale; however, these patterns do not hold consistently for all cancer types. This inconsistency might indicate that our current understanding of immune escape mechanisms is not entirely comprehensive, or it could suggest that immune evasion impact depends on the tissue type.

In summary, our escape classification, in conjunction with immune infiltration measures, seems to illustrate varying stages of co-evolution between somatic and immune cells, influencing cancer prognosis differently across various organs and tissues.

## Discussion

In this study, we compared the selective landscape of immune escaped versus non-escaped tumors using the ratio of nonsynonymous to synonymous mutations, dN/dS. Using a cohort stratification based on the mutational status of immune-related genes or “escape genes,” we discovered specific cancer drivers for each category and revealed new driver genes across tumor types in TCGA. We found that escaped tumors evolve more neutrally than non-escaped tumors when using cohort dN/dS of all genes and of known driver genes as a metric of selection. By analyzing the distribution of mutations at unique sites, we identified that mutations are more evenly distributed across driver genes in escape + patients. This finding suggests that the relaxation of immune selection on driver sites may result in a lower dN/dS ratio for known driver genes; however, this signal could also be attributed to a higher mutation accumulation observed in the escape group. Interestingly, a recent model [25] explains hotspots events in driver genes as a trade-off fitness between oncogenicity and immunogenicity, possibly confirming observed differences between mutation distribution on our escaped and non-escaped cohorts. As we could expect from our findings with the distribution of mutations, the proportion of mutational signatures also varies between escape + and escape − . Mutational signatures associated to immune evasion were associated to APOBEC and tobacco smoke, while the signatures associated to non-escaped were related to mismatch repair and chemical exposure. Overall, we see that our stratification strategy reveals new potential target genes and different evolutionary strategies. Our study sheds light on the evolutionary dynamics of tumor clones growing under immune surveillance, reflected by differences on mutational signatures and the mutational landscape of driver genes. Ultimately, escape status could also be used to determine the prognosis of patients depending on their mutational background and tumor microenvironment interactions.

The importance of the interplay between the immune system and cancer cells has been widely studied from a neoantigen and MHC-class I perspective [26–29]. A study by Grasso et al. observed the role of genetic mechanisms of immune evasion in colorectal cancer [30]. They focused on the type of genetic instability (e.g., microsatellite) rather than on mutations linked to an escape status. In our work, we could identify a prognostic value of the escape status specifically in immune inflamed patients. A result in line with a recent analysis of pancreatic cancer patients, where Łuksza et al. demonstrated that long-term survivors were subjected to strong immune editing, developed genetically less heterogeneous recurrent tumors with fewer neoantigens [27]. These observations are aligned with our findings highlighting the importance of understanding immune evasion mechanisms, selection strategies of tumor clones, and clinical outcomes.

Interestingly, driver events have a significantly higher clonality than mutations in escape genes, suggesting that the selective advantage given by driver events precedes immune escape. However, it is challenging to estimate the timing of escape mutations, and perhaps not all mutations in escape genes lead to a dysfunctional antigen presentation, especially as we did not consider the impact of copy number alterations affecting driver genes. Another caveat is that novel genes identified as drivers in the escape group may be byproducts of other functional drivers and differences on dN/dS could simply relate to increased antigenicity of well-expressed proteins or due to a trade-off between oncogenicity and immunogenicity of specific mutations, as suggested recently [25].

## Conclusions

Overall, we demonstrate key differences in the driver gene landscapes of escaped versus non-escaped tumors. We observed how immune evasion impacts the distribution of mutations along known driver genes, especially in hotspots associated to lower neoantigen presentation. We also observed that mutational signatures are associated to immune evasion mechanisms with tobacco signature being prevalent in escaped tumors. Interestingly, when considering immune categories and escape status, we observe better prognosis of immune escape only in immune-inflamed tumors suggesting that inflammation is a requisite for the emergence of immune evasion in cancer. Ultimately, our work highlights the importance of immune evasion as a cancer hallmark and its effect as a compensatory mechanism when negative selection forces, such as the one exerted by the immune system, are in action.

## Methods

### Dataset

We collated a comprehensive catalog of 88 genes implicated in the antigen presentation pathway or previously associated with immune evasion [18]. These genes, designated as “escape genes,” were detailed in Additional file 2: Table S1. A cohort of 9896 patients across 31 cancer subtypes from the TCGA database was stratified into escape + (with non-silent point mutations in escape genes) and escape − groups. The Cancer Genome Atlas (TCGA) project was accessed through Genomics Data Commons (GDC) Portal (https://portal.gdc.cancer.gov/) (13/10/2021). Patient number, tumor mutation burden, tumor types, and their respective acronyms are described in Additional file 2: Table S2.

For the immune category stratification, we obtained the groups C1 to C6 from Thorsson et al. 2018 assigned to each patient [26]. These are wound healing (C1), IFN-γ dominant (C2), inflammatory (C3), lymphocyte depleted (C4), immunologically quiet (C5), and TGF-β dominant (C6).

### Mutational pre-processing, escape classification and dN/dS calculation

We obtained the set of somatic mutations available on the TCGA VCF files without realignment to the reference as this will require recalling all variants from the original BAM files (see 1.1). We divided patients into escape + and escape − cohorts depending on whether they had a point mutation in one of the 88 “escape genes” [18]. We looked at the distribution of mutations in escaped genes by cancer type and performed hierarchical clustering on the genes and on the tumor types. We estimated tumor mutation burden for escape + and escape − , and then run the R package dNdScv (version 0.0.1.0) to calculate dN/dS (reference genome GRCh38) and detect positively selected genes. dndsCV employs a maximum likelihood estimate framework to calculate dN/dS along with corresponding 95% confidence intervals using a compilation of somatic mutations from multiple individuals (cohort analysis). This calculation can be tailored to single genes or specific lists of genes or encompass all genes. In the context of driver genes, dN/dS ratios were specifically calculated for a predefined set of 365 known driver genes (from ref. [4]). When referring to the global dN/dS ratio, we analyzed the entire set of 19,562 protein-coding genes on all patients, escape + and escape − groups. Significant driver genes for each group were obtained by selecting genes with dN/dS > 1 and adjusted *p*-value less than 0.1.

### Randomization analysis

We sampled a list of 88 genes 100 times randomly and assign patients into escape if they have a point mutation one of these genes. We excluded patients that were truly escape (a point mutation on the true set of escape genes). We ran dndsCV to obtain driver dN/dS from the randomized list and compare with our true set of “escape genes” list.

### Immunopeptidome—driver gene interactions

Building on our prior research [18], we sought to examine the interaction between the immunopeptidome and driver genes by excluding mutations within driver genes that intersect with the patient-specific immunopeptidome. To accomplish this, we retrieved the immunopeptidome data for all TCGA patients from SOPRANO [18]. Utilizing Ensembl biomaRt (version 2.56.0) available at Ensembl Biomart, we mapped the protein and gene names to their respective transcript ids. Subsequently, with the help of the IRanges package (version 2.34.0) and the ensembldb package (version 2.24.0), we translated these protein positions into genomic coordinates. This process allowed us to curate a filtered list by removing any mutations that overlapped with regions of the immunopeptidome. Finally, we conducted an analysis of the driver dN/dS ratios for both the filtered and original datasets.

### Stratification based on PDL1 expression

We obtain FPKM values expression data for each patient and all protein coding genes using the RTCGA package. We stratified patients based on whether they had low or high PD-L1, based on the normalized counts of the bottom 25% and top 25% respectively. This was done on all cancer types with available data.

### Stratification based on deletions in escape genes

We downloaded the CNV per gene from TCGA and got the sample_id of patients who had a deletion (CNV =  < 1) in the list of immune-related genes. We reclassified patients into 6 categories. Category A includes individuals with no evidence of escape at the level of point mutations or deletions. Category B includes patients with hemizygous deletions in at least one of the escape genes, no point mutations. Category C includes patients with homo or hemizygous deletions, but no point mutations. Category D includes patients with a point mutation or with a hemizygous deletion and category F includes patients with any mutation in one of the escape genes. Error bars indicate 95% confidence interval to the dN/dS estimate.

### Variant allele frequency

We obtained the variant allele frequency (VAF) for each mutation and compared the VAF distributions of driver and escape genes. We plot the VAF distributions and determine their statistical difference using Mann–Whitney test on the pancancer and the tumor type level.

### Mutation distribution for escape and driver genes

To compare the distribution of total mutations and unique mutated sites between escape + and escape − , we performed a chi-square test using ggstatsplot::ggbarstats (version 0.9.1). For this, we selected common driver genes (i.e., IDH1 and KRAS) and create contingency tables with the number of mutations and the number of unique mutated sites for escape + and escape − groups.

To account for a possible bias due to patient number differences or tumor mutational burden, we repeated the analysis by downsampling the number of patients or number of mutations to be the same between the two groups. We also repeated the analysis by excluding mutations occurring at the main hotspots of TP53, IDH1, KRAS, and HRAS. We used the R package pheatmap to cluster cancer types based on the number of escape mutations. We used cBioPortal to create lolliplots showing the different locations and types of mutations between escape + and escape − cohorts pancancer. We focused on the driver genes of our pancancer analysis. We also created lolliplots after removing mutational hotspots to compare the landscapes. To compare the distribution of escape + and escape − mutations in oncogenes and tumor suppressors, we obtained the annotation from the Catalogue of Somatic Mutations In Cancer, COSMIC. We assigned genes as either tumor suppressor or oncogenes if they contain TSG or Oncogene in the database, respectively. We then count the number of missense mutations falling in escape + and escape − groups.

### Mutational signatures

To find the most dominant signatures, we used the deconstructSigs (version 1.8.0) R package. This enables us to find mutational signature associated to each cohort. We then looked at the most frequent signatures and reported the 11 dominant signatures which occurred most often. This methodology was applied to the pancancer cohort and TCGA cancer types. We also plotted the proportion of mutations specific to the escape groups by running deconstructSigs on the escape + /escape − pancancer cohorts as a single sample. We tested whether the difference in the proportion of mutational signatures is statistically significant between the escaped cohorts using a chi-square test accounting for the number of mutations. Multiple test correction was performed using the Benjamini–Hochberg method.

### Statistics and data visualization

All statistical analyses were conducted using R statistical software (R v4.1.2), and *p*-values were adjusted for multiple comparisons where applicable using Benjamini Hochberg correction. All statistical analysis was performed using R. The ggplot2 (version 3.3.5) and ggpubr (version 0.4.0) packages were used for data visualization. Significant genes were selected based on dN/dS > 1 for missense mutations, after excluding olfactory receptors as they are spuriously mutated in cancer and unlikely to have a cancer driver role. For correlation, we used *r* Pearson and label the groups according to their escape status. We employed Wilcoxon-Mann tests to assess the differences in dN/dS ratios between escape + and escape − groups.

### Survival analysis

Clinical data was obtained from previous studies [31]. The data consisted of overall, disease-free, and progression-free survival data. We used the survminer R package (0.3.0) to determine the Kaplan Meier statistics and plot the differences on overall survival between groups. We applied this strategy to pancancer and to each tumor type to compare escape + and escape − groups under different scenarios.

### Supplementary Information

Additional file 1. The file contains supplementary figures (Fig S1-S23).

Additional file 2. The file contains supplementary tables (Tables S1-S6).

Additional file 3. Review history.

## Data Availability

Scripts for reproducing figures are available at Zenodo [32] (10.5281/zenodo.11489956). dNdScv package can be obtained from https://github.com/im3sanger/dndscv. SOPRANO package can be accessed on https://github.com/luisgls/SOPRANO.
