# Supplementary material for: Immune evasion impacts the landscape of driver genes during cancer evolution
Source: Genome Biol. 2024 Jun 26;25:168. doi: 10.1186/s13059-024-03302-x (PMC11210199; doi:10.1186/s13059-024-03302-x)
Supplement: Supplementary file 1 — Additional file 1. The file contains supplementary figures (Fig S1-S23). [file 13059_2024_3302_MOESM1_ESM.docx]

***Supplementary Figures***

***Immune evasion impacts the landscape of driver genes during cancer evolution.***


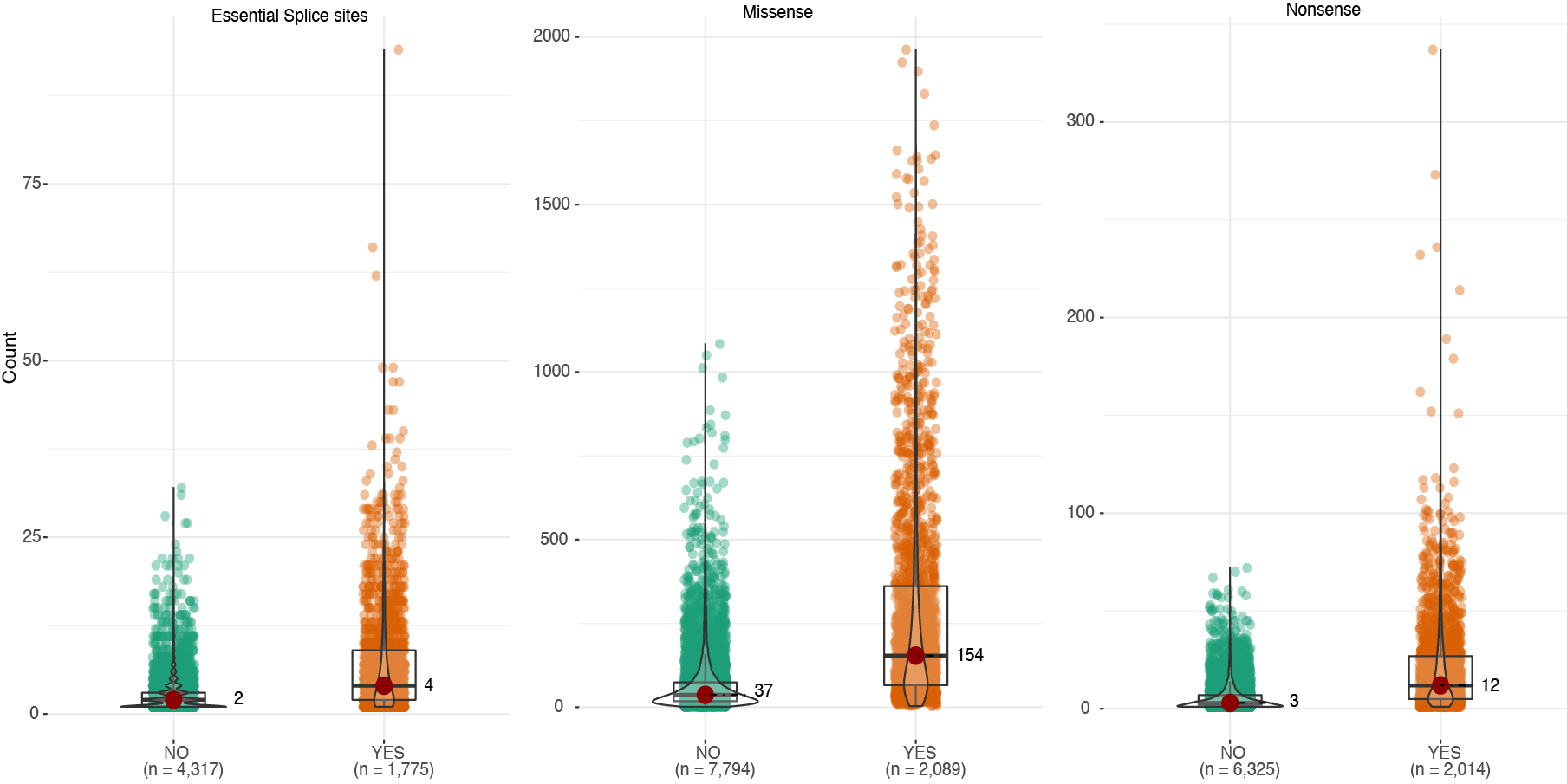


Count

Fig S1. Mutation burden for splice, missense and nonsense mutations in escaped+ (YES) and escaped- (NO) across all patients. Boxplots denote the median, 25th percentile, and 75th percentile, and whiskers are 1.5 times the interquartile range (IQR).

Fig S2. Bar plot with total number of patients per tumor type colored by escape status (light blue: Escaped-, dark blue: Escaped+).

Count

Patient frequency

Fig S3. Heatmap of mutated patient frequency in escape genes across TCGA tumor subtypes (Red indicates 50%).

dN/dS

Fig S4. Cohort dN/dS for all genes (global dN/dS) across all tumor types separated by point mutations (missense or truncating) in an escape gene (escaped- or NO, escaped+ or YES). Error bars indicate 95% confidence interval to the dN/dS estimate.

dN/dS

*Fig S5. Pancancer driver dN/dS on random escape genes (sampled 100 times).*

dN/dS

Fig S6. Cohort dN/dS for all genes (global dN/dS) for 31 cancer types separated by escape+ (blue) and escape- (red). Error bars indicate 95% confidence interval to the dN/dS estimate.

dN/dS

Fig S7. Cohort dN/dS for driver genes (driver dN/dS) across different categories of escape status. Category A includes individuals with no evidence of escape at the level of point mutations or deletions. Category B includes patients with hemizygous deletions in at least one of the escape genes, no point mutations. Category C includes patients with homo or hemizygous deletions, but no point mutations. Category D includes patients with a point mutation or with a hemizygous deletion and category F includes patients with any mutation in one of the escape genes. Error bars indicate 95% confidence interval to the dN/dS estimate.

dN/dS

Fig S8. Cohort dN/dS for driver genes (driver dN/dS) in all tumor types stratified by PDL1 expression (YES=high PDL1 expression, NO=low PDL1 expression). Error bars indicate 95% confidence interval to the dN/dS estimate.

Fig S9. Pancancer volcano plot of -log10(P value) versus log2(dN/dS) for truncating mutations in A) escaped+ and B) escaped- individuals.

Fig S10. dN/dS of 55 escaped- significant driver genes calculated for escaped- (red) and escaped+ (blue) individuals across Pancancer. Error bars indicate 95% confidence interval to the dN/dS estimate.

Fig S11. dN/dS of 17 escaped+ significant driver genes calculated for escaped- (red) and escaped+ (blue) individuals across Pancancer. Only two have a clear distance from dN/dS of one (CASP8 and SLITRK6). Error bars indicate 95% confidence interval to the dN/dS estimate.

Fig S12. dN/dS of 13 commonly (escaped+ and escaped-) significant driver genes calculated for escaped- (red) and escaped+ (blue) individuals across Pancancer. Four genes KRAS, FBXW7, NFE2L2 and SMAD4 have point estimates that overlap with the confidence interval. Error bars indicate 95% confidence interval to the dN/dS estimate.

Allele Frequency

Fig S13. Allele frequency of mutations in driver genes (green) and escape genes (orange) pancancer. Boxplots denote the median (red dot), 25th percentile, and 75th percentile, and whiskers are 1.5 times the interquartile range (IQR).

Allele Frequency

Fig S14. Allele frequency of mutations in driver (Red) and escape (Blue) genes shown for every cancer type. Boxplots denote the median, 25th percentile, and 75th percentile, and whiskers are 1.5 times the interquartile range (IQR).

Fig S15. Chi-square test of total mutations versus uniquely mutated sites by downsampling number of patients in A) IDH1 and B) KRAS genes.

Fig S16. Chi-square test without major hotspots in selected driver genes across pancancer patients.

*Fig S17. Chi-square test of escape+ versus escape- mutations in oncogenes and tumour suppressor genes.*

Fig S18. A) ER positive driver dN/dS on escape+ and escape- patients, B) ER negative driver dN/dS on escape+ and escape- patients from Breast cancer patients (BRCA).

Fig S19. A) HPV positive driver dN/dS on escape+ and escape- patients, B) HPV negative driver dN/dS on escape+ and escape- patients from Head and Neck cancer patients (HNSC).

**

Fig S20. Driver dN/dS on escape+ and escape- patients A) HBV positive, B) HBV negative, D) HCV positive, D) HCV negative from liver hepatocellular carcinoma (LIHC).

Fig S21. Chi-square test of mutational signature prevalence between escape+ and escape- groups for each tumor type. P-values are multiple test corrected using the Benjamini-Hochberg method.

Fig S22. Survival and proportion comparison for escape+ versus escape- patients stratified by immune categories from Thorsson et al. A: C1 – Wound healing, B:C2 – IFNg dominant, C: C3 –Inflammatory, D: C4 – Lymphocytes depletion, E: C5 – Immunological quiet, F:C6 – TGFb dominant.

Fig S23. Survival for escape+ versus escape- of specific cancer types. A:BLCA, B: CESC, C: MESO, D: THCA, E: THYM.
