# Supplementary material for: Immune evasion impacts the landscape of driver genes during cancer evolution
Source: Genome Biol. 2024 Jun 26;25:168. doi: 10.1186/s13059-024-03302-x (PMC11210199; doi:10.1186/s13059-024-03302-x)
Supplement: Supplementary file 3 — Additional file 3. Review history. [file 13059_2024_3302_MOESM3_ESM.docx]

Review History

**First round of review**

**Reviewer 1**

**Are you able to assess all statistics in the manuscript, including the appropriateness of statistical tests used?** Yes, and I have assessed the statistics in my report.

**Comments to author:**

Summary: Gourmet et al explore the relationship between immune escaped tumors and the driver landscape, mutational signatures and disease prognosis. Immune escape is inferred based on the mutational status of 88 genes. They find some bias in driver gene mutations in escaped vs non-escaped tumors, as well as differences in mutational signature and survival outcome specifically in the inflamed context. Overall, the analysis is interesting, and the finding that driver mutations are under less constraint to accumulate mutations in immune escaped tumors is thought provoking. However, there are some weaker aspects. For some of the analyses, it isn't always clear what is due to immune escape versus the overrepresentation of particular tumor types with specific mutational characteristics in immune escaped vs non-escaped groups. It was also not clear how effectively the mutation status at the 88 genes grouped tumors into immune escaped vs non-escaped groups.

Major Comments:

The designation of escape+ and escape- is done on the basis of mutation status at a set of 88 genes in the antigen presentation pathway. Escape+ tumors are those with a nonsynonymous mutation, and these tumors are also observed to have a higher overall mutation burden. It was not clear if copy number alterations were considered, or whether higher variant allelic fraction in the DNA was important. Is there any indication that some tumors escape early versus late? The CNV aspect is potentially addressed on line 114 but it was not entirely clear from the description how many tumors would be reclassified as escape+ when deletions are included and how the statistics change, or which definition was used for the remainder of the manuscript.

Furthermore, individuals with a higher mutation/CNV burden may also be more likely to have a mutation/CNV in the set of 88 genes by chance. In their prior work, the authors used randomization to show that immune dN/dS is more extreme when mutations are present in one of these 88 genes than for a random selection of 88 genes. It would be appropriate to demonstrate this again here.

Finally, it appears that class I HLA genes were included in the 88 genes (and possibly also class II?) but it was unclear whether missense mutations were called relative to the reference genome or the individuals HLA alleles. Because HLA is highly polymorphic, it is inappropriate to call missense variants relative to the reference genome. Instead, a tool like Polysolver should be used to call them relative to the patient's own HLA alleles. It was unclear from the methods whether this was the case.

In their prior work, the authors focused on dN/dS in regions of the proteome that could be presented by an individual's MHC alleles, the so called immune dN/dS. This manuscript appears to have deviated from focus on the regions where mutations are more likely to be immunogenic. The reason for this was not entirely clear. It was also unclear whether expression of mutations was considered. Relating to the main conclusion, that immunoediting constrains the dN/dS of drivers, is this attributable to immune surveillance against MHC exposed regions of driver proteins, or something else?

How does the author's definition of escape+ and escape- compare to tumor designation as immune infiltrated, excluded or cold?

When stratifying by escape+/-, tumor type and specific driver genes, the number of mutations is likely to become relatively small. The authors need to be transparent about the number of datapoints used to infer positive or negative selection for driver mutations. In addition, some driver events occur through copy number variants or structural variants rather than point mutations. It was unclear whether these were considered.

The authors write "We then tested all significant common driver genes together and the majority of cancer drivers harbored mutations at specific sites in escape- patients (hotspots), while mutations where evenly distributed across the gene in escape+ patients (Fig 3G, Pandriver p-value= 2.49e−53). This was also the case for other known driver genes such as BRAF (p-value =3.01E-08), TP53 (p-value =3.41E-11), EGFR (p-value = 0.00877) and GNA11 (p-value =0.00689). " It is well established that oncogenes tend to harbor mutations at a limited number of sites that increase protein activity, whereas tumor suppressor genes tend to have a broader distribution of mutated sites. Was there a bias for escape+ patients to harbor more mutations in tumor suppressors than oncogenes? Furthermore, not all mutations in oncogenes and tumor suppressors are necessarily driver mutations, and the selective advantage of a mutation in a driver gene depends on the presence or absence of other driver events in related genes in the same cells. Thus, while the data could suggest that immune selection limits the availability of driver sites, as the authors imply, it could also suggest that immune selection is less effective in tumors with a driver mutation at a hotspot in these genes, or more effective when other genes are driving the tumor growth. This could be investigated in more depth.

For the mutational signature analysis in Fig 4E, it is unclear that the differences in mutational signature reported are directly associated with immune escape or simply the distribution of specific tumor types across the immune escaped categories. Did the authors control for tumor type composition of each group? They did compare within HNSCC to observe that immune escape was more likely in the presence of a mutagenic exposure - does this hold for other

The authors should consider evaluating certain subtypes of cancer separately, for example ER+ versus ER- breast cancer, as ER- tumors have been found to be much more immune infiltrated and also have an enrichment for TP53 mutations relative to ER+ breast cancers. Similarly, it may be worth considering separately HNSC and LIHC tumors that are virally driven versus not.

The survival analysis is interesting, but different tumor types should not be mixed in a Kaplan Meier analysis. Some tumor types have long survival times while others are diagnosed late and kill quickly, so any nonrandom assortment of tumor type to escaped+ vs - could create the appearance of a survival difference that is not actually associated with immune escape. At the very least, a CoxPH model with tumor type explicitly modeled should be considered. However, it would be better to revisit this within tumor type.

Minor Comments:

Though the inferred relative timing is unlikely to change, the authors should consider using cancer cell fraction instead of variant allelic fraction to control for tumor purity and local copy number alterations when inferring the relative timing of variants.

It could be pretty interesting to revisit the driver analysis considering the timeing of events using the algorithms for dissecting the molecular archaeology of cancer generated by the Van Loo group at MD Anderson.

The also authors mention work by Luszka in pancreatic cancer that shows that immunoedited pancreatic cancers show less intratumoral heterogeneity (this has also been seen in mice). It might be interesting to compare intratumoral heterogeneity vs immune escape status in various tumor types.

The authors should include the requested statement about using the TCGA dataset in the acknowledgements section (see TCGA website).

106 / 107 - this statement is confusing. Did the authors mean that out of 31 different tumor types, the aggregate dN/dS ratio was greater than 1 for more often for escape- vs escape+ tumors?

Line 123 - not a complete sentence

Line 202 - run -> ran

Line 209-211 - awkward grammar. rephrase

Lines 216 and 219 both start with 'interestingly'

Line 243 - worst -> worse

Line 393 - Zapata Nature Genetics reference appears to need to be updated

Line 444- dnds -> dN/dS

**Reviewer 2**

**Are you able to assess all statistics in the manuscript, including the appropriateness of statistical tests used?** Yes, and I have assessed the statistics in my report.

**Comments to author:**

The manuscript "Immune evasion impacts the landscape of driver genes during cancer evolution" investigates how the immune systems impacts the landscape of driver gene mutations in tumors. The authors analyze a set of 9896 primary tumors from TCGA, coming from various types of cancer looking at the dN/dS measure, the ratio of nonsynonymous to synonymous mutations. The dN/dS ratio has been originally developed for application to distantly diverged sequences, the differences among which represent substitutions that have fixed along independent lineages. It has since been applied for analysis of cancer data.

The authors have classified the tumors into two groups: escape+ and escape-, based on their mutations in a predefined set of 88 immune-related genes. 85 out of these genes where then identified as driver genes. After grouping of patients into the 2 categories, the authors identified the sets of genes that have significantly high dN/dS in each of the groups. They also observe more uniform distribution of the mutations in drivers genes of the escape+ group as opposed to escape- group.

Interestingly, the authors identified different mutational signatures in the two sets of patients.

Finally, the authors checked if the classification into the escape+ and escape- group is related to a better survival in the escape- group. No such signal was observed on the entire dataset, however additional classification of tumors into 6 immune categories revealed one category - "inflammatory signature" with a significant separation.

The manuscript presents interesting results supporting the hypothesis that immune selection shapes tumor evolution and that it is an import factor determining tumor fitness. However, the manuscript requires some clarifications in the analysis:

Major comments:

1. The exact steps of the analysis are at places unclear. It is difficult to deduce which set of genes is used at which point. There appear to be 3 distinct sets used: (1) the set of immune-related genes, (2) the set of known tumor driver genes and (3) the set of de novo defined driver genes (e.g. line 99 - what are all genes? line 122 - does it refer to dN/dS on the immune-related genes?).

2. It should be clarified in the methods how dN/dS is computed: is it computed for each patient on a given set of genes and the reported values and variances/confidence intervals are over the set of individuals? Or is there some other approach? In particular, what are the error bars in Fig 1A and 1B and how is the single dN/dS value per gene derived, as reported in Fig 1C and 1D, 1H.

3. Line 187-192: the text and conclusions are unclear. What does it mean that selection restricts availability of driver sites, availability in what sense?

"By removing mutations in hotpots of driver genes" - which and how many mutations were removed and how are hotspots defined?

4. Mutational signatures - accounting for the number of mutations, are the differences in the mutational signatures significant?

5. Survival analysis:

Is the separation similar within the cancer types? What is the number of tumors contributing to the survival analysis in Fig. 5?

Minor comments:

1. Sentence in line 123 lacks the predicate

2. dNdS should be dN/dS in lines 173-174.

3. Captions and labels on the plots have too small font and are hardly readable.

**Authors Response**

**Point-by-point responses to the reviewers’ comments:**

Revision genome biology

Reviewer reports:

*Reviewer #1: Summary: Gourmet et al explore the relationship between immune escaped tumors and the driver landscape, mutational signatures and disease prognosis. Immune escape is inferred based on the mutational status of 88 genes. They find some bias in driver gene mutations in escaped vs non-escaped tumors, as well as differences in mutational signature and survival outcome specifically in the inflamed context. Overall, the analysis is interesting, and the finding that driver mutations are under less constraint to accumulate mutations in immune escaped tumors is thought provoking. However, there are some weaker aspects. For some of the analyses, it isn't always clear what is due to immune escape versus the overrepresentation of particular tumor types with specific mutational characteristics in immune escaped vs non-escaped groups. It was also not clear how effectively the mutation status at the 88 genes grouped tumors into immune escaped vs non-escaped groups.* 

We thank the reviewer for such thoughtful reviews, we have made our best effort to address all the comments and have substantially improved the manuscript. We hope that this new version with all the additional analyses is suitable for publication.

Major Comments: 

The designation of escape+ and escape- is done on the basis of mutation status at a set of 88 genes in the antigen presentation pathway. Escape+ tumors are those with a nonsynonymous mutation, and these tumors are also observed to have a higher overall mutation burden. It was not clear if copy number alterations were considered, or whether higher variant allelic fraction in the DNA was important. Is there any indication that some tumors escape early versus late? The CNV aspect is potentially addressed on line 114 but it was not entirely clear from the description how many tumors would be reclassified as escape+ when deletions are included and how the statistics change, or which definition was used for the remainder of the manuscript.

Response: It should be noted that in our initial analyses, copy number alterations within the escape gene set were not considered, due to the speculative nature of their impact on immune evasion (for instance, whether a copy number gain of B2m is advantageous or disadvantageous for escape remains unclear). However, we have incorporated copy number profiles for these 88 genes and categorized patients into six groups based on the presence of point mutations or deletions. Our findings indicate that the driver dN/dS ratio approximates one (suggesting neutrality) in the presence of either a deletion or a point mutation within this gene set, and deviates from one in their absence (Response Fig. 1). Consequently, tumors with escape mutations appear to evolve more neutrally compared to their non-escape counterparts, reinforcing our previous findings.

To determine the temporal aspect of the escape phenotype, we compared the variant allelic fraction (VAF) of escape genes against a random gene set (Response Fig. 2). Such analysis revealed no significant difference in VAF between escape and non-escape genes, implying that the escape phenomenon is more likely a later event in tumor evolution. Additionally, we wish to highlight our recent preprint (Gourmet et al. 2024, https://www.biorxiv.org/content/10.1101/2024.01.21.576566v1) that addresses the sequential emergence of cancer hallmarks, including immune inflammation and escape. In this novel study we identified escape genes as being subject to positive selection but exhibiting lower VAF relative to other hallmark genes.

dN/dS

*Figure 1. Cohort dN/dS for driver genes across different categories of escape status. Category A includes individuals with no evidence of escape at the level of point mutations or deletions. Category B includes patients with hemizygous deletions in at least one of the escape genes, no point mutations. Category C includes patients with homo or hemizygous deletions, but no point mutations. Category D includes patients with a point mutation or with a hemizygous deletion and category F includes patients with any mutation in one of the escape genes. Error bars indicate 95% confidence interval to the dN/dS estimate.*

*Figure 2. VAF of escape genes versus random gene across multiple tumor types.*

*Furthermore, individuals with a higher mutation/CNV burden may also be more likely to have a mutation/CNV in the set of 88 genes by chance. In their prior work, the authors used randomization to show that immune dN/dS is more extreme when mutations are present in one of these 88 genes than for a random selection of 88 genes. It would be appropriate to demonstrate this again here.*

Response: We thank the reviewer for this suggestion. To account for potential bias due to an elevated mutation/CNV burden, we implemented a randomization strategy, repeating the process 100 times with an alternate set of 88 genes. In this revised analysis, patients with nonsynonymous mutations in the original set of escape genes were excluded to avoid confounding effects. The results demonstrate that the driver dN/dS ratio for the randomized escape+ cohort (right plot, approximately 1.5) was higher than that observed in the true set of escape+ patients, aligning closely with the escape- group's ratio (Response Figure 3).

*Figure 3. Driver dN/dS value for a patients having a mutation in a random set of 88 genes.*

*Finally, it appears that class I HLA genes were included in the 88 genes (and possibly also class II?) but it was unclear whether missense mutations were called relative to the reference genome or the individuals HLA alleles. Because HLA is highly polymorphic, it is inappropriate to call missense variants relative to the reference genome. Instead, a tool like Polysolver should be used to call them relative to the patient's own HLA alleles. It was unclear from the methods whether this was the case.*

Response: We acknowledge the reviewer's concern regarding the identification of missense mutations in the highly polymorphic HLA genes. As we did not access the TCGA bam files, we could not realign reads to the personal reference genome. We agree that the best strategy to call accurately somatic missense or any other mutations from highly polymorphic regions is to run tools such as LILAC (Martinez-Jimenez et al., 2023, <https://www.nature.com/articles/s41588-023-01367-1>) that benefit from tumor normal paired bam files. As rightly the reviewer indicates, Mutect somatic calls could be false positive, and patients can be falsely classified as escape. Thus, our approach is highly conservative as including truly selected patients (considered escaped when they aren’t) will push driver dN/dS far above one.

We must note that if mutations in HLA genes were false positives or artifacts, they would not exhibit signs of positive selection using dN/dS. Conversely, if these mutations were unfiltered germline polymorphisms, we would expect to observe a signal indicative of negative selection, as demonstrated in prior studies (Martincorena et al., 2017). However, in our recent publication (Zapata et al., 2023), and corroborated by other research (Martinez-Jimenez et al., 2023), there is strong evidence of positive selection in these genes.

In their prior work, the authors focused on dN/dS in regions of the proteome that could be presented by an individual's MHC alleles, the so called immune dN/dS. This manuscript appears to have deviated from focus on the regions where mutations are more likely to be immunogenic. The reason for this was not entirely clear. It was also unclear whether expression of mutations was considered. Relating to the main conclusion, that immunoediting constrains the dN/dS of drivers, is this attributable to immune surveillance against MHC exposed regions of driver proteins, or something else?

Response: Thank you for highlighting these crucial aspects. In our prior research (Zapata et al., 2023), we evaluated immune selection strength without regard to escape status. Noticing that escape status modulated selection within MHC-exposed regions, we investigated whether it similarly affected signals of positive selection in driver genes. In Figure 4G of Zapata et al., 2023, we initially assessed driver dN/dS ratios by both therapy response and escape status within a subset of patients receiving immunotherapy. Our findings indicated a loss of positive selection signal in driver genes for escape+ patients, prompting us to explore this further in a separate study.

To address the impact of immune surveillance against MHC-exposed regions on driver dN/dS, we performed an analysis where all nonsynonymous mutations within the immunopeptidome (spanning 70 HLA alleles) were masked, after which we recalculated the driver dN/dS ratios for both escape+ and escape- patient groups. This analysis revealed that for escape- patients, driver dN/dS was significantly more impacted when considering removing immunopeptidome mutations, as opposed to escape+ patients for whom the difference between filtered and unfiltered driver dN/dS ratios was negligible. This corroborates that in patients who have escaped, the immune system's selective pressure on driver dN/dS is no longer present (Response Fig. 4).

Top of Form

Bottom of Form

dN/dS

*Figure 4. Driver dN/dS with mutations in the immunopeptidome filtered or unfiltered for escape- (left) and escape+ (right).*

How does the author's definition of escape+ and escape- compare to tumor designation as immune infiltrated, excluded or cold?

Response: We have adopted the classification framework from Thorsson et al., 2017, which delineates six clusters including inflammatory, lymphocyte-depleted, and immunologically quiet categories, among others. Upon examination, our escape+ and escape- categories did not show significant enrichment within these clusters, with the notable exception of category C5 – the immunologically quiet group (Response Fig. 5). This category was predominantly composed of escape- patients, suggesting that an active immune system is necessary to generate escaped patients.

*Figure 5. Proportion of patients classified as escape+ or escape- for Thorsson categories.*

When stratifying by escape+/-, tumor type and specific driver genes, the number of mutations is likely to become relatively small. The authors need to be transparent about the number of datapoints used to infer positive or negative selection for driver mutations. In addition, some driver events occur through copy number variants or structural variants rather than point mutations. It was unclear whether these were considered. 

Response: We thank the reviewer for this pertinent observation. In determining driver genes, we applied the model developed in dNdScv (Martincorena et al 2017), which relies on point mutations to estimate dN/dS per gene and statistical significance following multiple test correction. The number of mutations used for all analysis is described in supplementary table 2. Additionally, as we have previously noted, due to the absence of established models for calculating selective coefficients for copy number alterations, these types of alterations were not included in our analysis of driver genes. This limitation has been acknowledged in the discussion section of our manuscript.

The authors write "We then tested all significant common driver genes together and the majority of cancer drivers harbored mutations at specific sites in escape- patients (hotspots), while mutations where evenly distributed across the gene in escape+ patients (Fig 3G, Pandriver p-value= 2.49e−53). This was also the case for other known driver genes such as BRAF (p-value =3.01E-08), TP53 (p-value =3.41E-11), EGFR (p-value = 0.00877) and GNA11 (p-value =0.00689). " It is well established that oncogenes tend to harbor mutations at a limited number of sites that increase protein activity, whereas tumor suppressor genes tend to have a broader distribution of mutated sites. Was there a bias for escape+ patients to harbor more mutations in tumor suppressors than oncogenes?

Response: To investigate potential biases related to the category of driver genes (oncogenes versus tumor suppressors), we categorized them based on the IntOGen database ([www.intogen.org](http://www.intogen.org/)). We found that missense mutations were evenly distributed between escape groups in tumor suppressor genes but not in oncogenes. Escape- patients tend to have slightly more mutations in oncogenes, similarly to what we found in the per gene analysis. These findings suggest that immune surveillance select against non-hotspot sites, supporting the overall conclusions of our study.

*Figure 6. Chi-square test of escape+ versus escape- mutations in oncogenes and tumour suppressor genes.*

Furthermore, not all mutations in oncogenes and tumor suppressors are necessarily driver mutations, and the selective advantage of a mutation in a driver gene depends on the presence or absence of other driver events in related genes in the same cells. Thus, while the data could suggest that immune selection limits the availability of driver sites, as the authors imply, it could also suggest that immune selection is less effective in tumors with a driver mutation at a hotspot in these genes, or more effective when other genes are driving the tumor growth. This could be investigated in more depth.

Response: Thank you for the valuable perspective. Indeed, our initial hypothesis assumes that immune selection may constrain the availability of driver sites, potentially due to the immunogenicity of mutations at these sites. However, we acknowledge the possibility that the oncogenic potential of certain mutations could outweigh the effects of immune selection. In essence, the fitness of a cancer cell may be a balance of oncogenicity against immunogenicity. Future experimental work will aim to tease apart these two influences. Such studies would ideally involve inducing identical mutations in different cellular contexts (immune-deficient versus immune-competent). We have added the following to the discussion: “Another caveat is that novel genes identified as drivers in the escape group may be byproducts of other functional drivers and differences on dN/dS could simply relate to increased antigenicity of well-expressed proteins or due to a trade-off between oncogenicity and immunogenicity of specific mutations, as suggested recently (25).”

For the mutational signature analysis in Fig 4E, it is unclear that the differences in mutational signature reported are directly associated with immune escape or simply the distribution of specific tumor types across the immune escaped categories. Did the authors control for tumor type composition of each group? They did compare within HNSCC to observe that immune escape was more likely in the presence of a mutagenic exposure - does this hold for other

Response: We regret the oversight in our initial explanation. To clarify, we did perform a comparative analysis of all mutational signatures across escape groups using a chi-square test (Response Fig 7). We noted that after multiple test correction our initial statement specific to HNSC was no longer significant, but it remained strongly significant for LUAD. We have corrected this in the manuscript. To better communicate our results, we have now included the following statement: "We also investigated differences in mutational signatures between escape groups per cancer type (Fig S21) and found that 16 out of 31 tumor types have at least one mutational signature significantly different." This addition aims to address the direct association of mutational signatures with immune escape across diverse tumor types.

*Figure 7. Chi-square test of mutational signature prevalence between escape+ and escape- groups for each tumor type. P-values are multiple test corrected using Benjamini-Hochberg correction.*

The authors should consider evaluating certain subtypes of cancer separately, for example ER+ versus ER- breast cancer, as ER- tumors have been found to be much more immune infiltrated and also have an enrichment for TP53 mutations relative to ER+ breast cancers. Similarly, it may be worth considering separately HNSC and LIHC tumors that are virally driven versus not.

Response: In response to the suggestion, we have conducted separate analyses for the cancer subtypes and incorporated our findings into the manuscript. We differentiated between ER+ and ER- breast cancers (Response Fig 8) and observed that the disparities between the escape groups were more pronounced in ER+ cases. In head and neck squamous cell carcinoma (HNSC, response Fig 9), we noted distinct patterns when comparing HPV+ to HPV- tumors: escape+ patients exhibited a higher driver dN/dS than escape- in HPV+ cases, whereas the trend was reversed in HPV- cases. Similarly, in liver hepatocellular carcinoma (LIHC) patients with HBV or HCV (Response fig 10), we identified a pattern analogous to that observed in HPV+ tumors. These observations imply that viral-driven tumors experience immune selective pressures, which impact driver dN/dS.

*Figure 8. A) ER positive driver dN/dS on escape+ and escape- patients, B) ER negative driver dN/dS on escape+ and escape- patients from Breast cancer patients (BRCA).*

*Figure 9. A) HPV positive driver dN/dS on escape+ and escape- patients, B) HPV negative driver dN/dS on escape+ and escape- patients from Head and Neck cancer patients (HNSC).*

*Figure 10. Driver dN/dS on escape+ and escape- patients A) HBV positive, B) HBV negative, D) HCV positive, D) HCV negative from liver hepatocellular carcinoma (LIHC).*

The survival analysis is interesting, but different tumor types should not be mixed in a Kaplan Meier analysis. Some tumor types have long survival times while others are diagnosed late and kill quickly, so any non-random assortment of tumor type to escaped+ vs - could create the appearance of a survival difference that is not actually associated with immune escape. At the very least, a Cop model with tumor type explicitly modeled should be considered. However, it would be better to revisit this within tumor type.

Response:

We acknowledge the concern that combining different tumor types can confound survival analysis. Originally, our analysis was intentionally designed to be independent of tumor type to assess the survival impact of our molecular classification. However, in response to the reviewer’s feedback, we have now included the proportion of patients from each tumor type in our calculations to better account for potential prognostic differences.

Upon re-evaluating the data with this additional stratification, we found that all tumor types are represented in both escape+ and escape- categories. Focusing on the specific impact of escape status within individual tumor types, we identified five tumor types with significant differences in survival outcomes.

We have updated the manuscript with the following statement to reflect these findings:

“Despite these global prognostic differences between escape+ and escape- classifications, such distinctions are not consistent at the individual tumor level. This suggests that our mapping of escape mechanisms may be incomplete, or that diverse evolutionary paths related to immune evasion might have varying prognostic impacts depending on the tissue type.”

*Figure 11. Survival for escape+ versus escape- of specific cancer types. A:BLCA, B: CESC, C: MESO, D: THCA, E: THYM.*

Minor Comments: 

Though the inferred relative timing is unlikely to change, the authors should consider using cancer cell fraction instead of variant allelic fraction to control for tumor purity and local copy number alterations when inferring the relative timing of variants. 

Response: We agree with the reviewer that the cancer cell fraction would indeed serve as an optimal metric for assessing the clonality/timing of somatic variants. As integrating this metric would introduce a layer of complexity that diverges from the primary objective of our current study, we aimed at exploring CCF impact on the timing in a separate study (Gourmet et al 2023). In short, using CCF instead of VAF has a subtle impact on the order of cancer hallmark acquisition but immune evasion remained the last to be acquired.

It could be pretty interesting to revisit the driver analysis considering the timing of events using the algorithms for dissecting the molecular archaeology of cancer generated by the Van Loo group at MD Anderson. 

Response: Thank you for this insightful suggestion. We concur that the timing of events offers a compelling avenue for research. As we noted previously, we have expanded this suggestion to analyse the sequence of cancer hallmark events in a separate manuscript, where variant allelic fractions (VAF) and cancer cell fractions (CCF) are considered. The focus of our current study is to delineate selective advantages by examining only dN/dS ratios in driver genes, deliberately independent of timing considerations.

The also authors mention work by Luszka in pancreatic cancer that shows that immunoedited pancreatic cancers show less intratumoral heterogeneity (this has also been seen in mice). It might be interesting to compare intratumoral heterogeneity vs immune escape status in various tumor types. 

Response: The findings by Luszka et al. show that immune-edited pancreatic tumors exhibit reduced intratumoral heterogeneity, mirroring the patterns we have observed; non-escaped patients have lower mutation burden overall, and evidence of strong immune selection in a broader repertoire of sites. In contrast, immune-escaped tumors display larger mutation burden, more significant driver genes, and more neutral dN/dS values, indicating a pattern that resembles more neutrally evolving tumors (absence of selection).

Top of Form

Bottom of Form

The authors should include the requested statement about using the TCGA dataset in the acknowledgements section (see TCGA website).

Response: We have included the required acknowledgment in our manuscript as follows: “The results shown herein are based upon data generated by the TCGA Research Network (https://www.cancer.gov/tcga).” This statement has been added to the acknowledgments section to ensure proper attribution and compliance with data usage policies.

106 / 107 - this statement is confusing. Did the authors mean that out of 31 different tumor types, the aggregate dN/dS ratio was greater than 1 for more often for escape- vs escape+ tumors?

Response: Thank you for pointing out the need for clarification, and yes, the reviewer is correct. We have revised the sentence for better clarity as follows: “When looking at each tumor subtype, global dN/dS was significantly higher than one in 25 out of 31 cancer types in escape- patients compared to 19 out of 31 tumor types in escape+ patients (Fig S6).” As now explained in the manuscript we refer to global dN/dS to the overall ratio of nonsynonymous to synonymous mutations on the protein sequence of ~20K protein coding genes. This suggest that overall the signal of positive selection is stronger in non-escaped genes.

Line 123 - not a complete sentence

Line 202 - run -> ran

Line 209-211 - awkward grammar. rephrase

Lines 216 and 219 both start with 'interestingly'

Line 243 - worst -> worse

Line 393 - Zapata Nature Genetics reference appears to need to be updated

Line 444- dnds -> dN/dS

Response: We thank the reviewer for these corrections. We have amended all of them. We sincerely hope that our efforts on improving the manuscript can move forward the field.

*Reviewer #2: The manuscript "Immune evasion impacts the landscape of driver genes during cancer evolution" investigates how the immune systems impacts the landscape of driver gene mutations in tumors. The authors analyze a set of 9896 primary tumors from TCGA, coming from various types of cancer looking at the dN/dS measure, the ratio of nonsynonymous to synonymous mutations. The dN/dS ratio has been originally developed for application to distantly diverged sequences, the differences among which represent substitutions that have fixed along independent lineages. It has since been applied for analysis of cancer data.

The authors have classified the tumors into two groups: escape+ and escape-, based on their mutations in a predefined set of 88 immune-related genes. 85 out of these genes where then identified as driver genes. After grouping of patients into the 2 categories, the authors identified the sets of genes that have significantly high dN/dS in each of the groups. They also observe more uniform distribution of the mutations in drivers genes of the escape+ group as opposed to escape- group.

Interestingly, the authors identified different mutational signatures in the two sets of patients.
Finally, the authors checked if the classification into the escape+ and escape- group is related to a better survival in the escape- group. No such signal was observed on the entire dataset, however additional classification of tumors into 6 immune categories revealed one category - "inflammatory signature" with a significant separation.

The manuscript presents interesting results supporting the hypothesis that immune selection shapes tumor evolution and that it is an import factor determining tumor fitness. However, the manuscript requires some clarifications in the analysis:*

Major comments:

1.      The exact steps of the analysis are at places unclear. It is difficult to deduce which set of genes is used at which point. There appear to be 3 distinct sets used: (1) the set of immune-related genes, (2) the set of known tumor driver genes and (3) the set of de novo defined driver genes (e.g. line 99 - what are all genes? line 122 - does it refer to dN/dS on the immune-related genes?).

Response: We appreciate the reviewer's attention to detail and apologize for any confusion arising from the gene lists presented. To clarify, we categorize genes into three distinct sets: 1) all protein-coding genes, comprising 19,562 genes in total; 2) a known set of 366 driver genes; and 3) immune-related or "escape” genes as we have recently defined them in Zapata et al 2023. The term "global dN/dS" specifically refers to the aggregated dN/dS ratio across all protein-coding genes. This strategy was used to detect novel driver genes in specific groups. When calculating dN/dS for driver genes, we exclusively use the known set of driver genes. To clarify, the reported driver dN/dS calculation was based on the list of known driver genes. We have revised the manuscript to explicitly define these terms and have detailed our methodology correspondingly.

2.      It should be clarified in the methods how dN/dS is computed: is it computed for each patient on a given set of genes and the reported values and variances/confidence intervals are over the set of individuals?  Or is there some other approach? In particular, what are the error bars in Fig 1A and 1B and how is the single dN/dS value per gene derived, as reported in Fig 1C and 1D, 1H.

Response: We apologize for any confusion caused and thank you for prompting a clarification on our methods for computing dN/dS ratios. We have updated the manuscript to include a more detailed explanation: "dN/dS ratios were calculated on cohort levels using the dndsCV package, which employs maximum likelihood estimation to determine dN/dS along with corresponding 95% confidence intervals, using a compilation of somatic mutations from multiple individuals. This calculation can be tailored to individual genes, specific lists of genes, or encompass all genes. In the context of driver genes, dN/dS ratios were specifically calculated for a predefined set of 365 known driver genes. When referring to the global dN/dS ratio, we analyzed the entire set of 19,562 protein-coding genes."

3.      Line 187-192: the text and conclusions are unclear. What does it mean that selection restricts availability of driver sites, availability in what sense? 
"By removing mutations in hotpots of driver genes" - which and how many mutations were removed and how are hotspots defined?

Response: In response to the feedback, we have refined our methods section to detail our analytical approach more accurately. Specifically, we addressed the potential confounding effect of the different numbers of escape- individuals by equalizing the cohort sizes through downsampling (Response Fig 12). This analysis revealed that for IDH1, the pattern of mutation distribution—both in terms of unique sites and total mutation count—persisted despite the adjustment, thus supporting the notion that immune selection may indeed influence the landscape of driver mutations.

Regarding hotspots, we identified and excluded mutations from the most frequently mutated sites within IDH1 and KRAS genes, namely R132 and G12. This strategy aimed to assess if these presumed non-immunogenic hotspots were skewing our observations. The analysis indicated no significant change for IDH1 (p-value: 0.13), which suggests that R132 may represent a hotspot that is inherently less recognized by the immune system. Conversely, for KRAS, the exclusion of G12 maintained the significant difference in mutation pattern, hinting at the potential relevance of additional hotspots in this gene (Response Fig 13).

*Figure 12. Chi-square test of total mutations versus uniquely mutated sites by downsampling number of patients in A) IDH1 and B) KRAS genes.*

*Figure 13.* *Chi-square test without major hotspots in selected driver genes across pancancer patients.*

4.      Mutational signatures - accounting for the number of mutations, are the differences in the mutational signatures significant?

Response: We thank the reviewer for this comment. We have repeated our analysis to account for the number of mutations for pancancer. We found that 46 out of 49 signatures as significant pancancer (Supp Table 5).

5.      Survival analysis: 
Is the separation similar within the cancer types? What is the number of tumors contributing to the survival analysis in Fig. 5?

Response:

When looking at specific cancer types, 5 of them displayed a significant difference in survival based on escape status only (not considering Thorsson immune category): BLCA, CESC, MESO, THYM and THCA. When looking at specific Thorsson immune categories, only SARC showed survival difference when focusing on immune category C3 (inflammatory). There were 2151 tumours contributing to the survival analysis in Fig 5, so we added a supplementary figure alongside the other pancancer survival Thorsson immune categories.

Minor comments:
1.      Sentence in line 123 lacks the predicate.

We thank the reviewer for noticing this. We have modified the manuscript accordingly.
2.      dNdS should be dN/dS in lines 173-174.

We thank the reviewer for noticing this. We have modified the manuscript accordingly.
3.      Captions and labels on the plots have too small font and are hardly readable.

We have adapted the figures following the journal guidelines.

**Second round of review**

**Reviewer 1**

The authors have addressed my comments.

Comments to Author:

I have a couple of minor additional suggestions.

Line 146: "Among 55 escape- specific genes, the majority (41/55) was evolving neutrally in the" -> "...the majority were evolving neutrally..."

Line 153: "Few genes were strong drivers in one group and fully neutral in the other, among drivers in escape-, GTF2I,155

VHL, REG1B, SPANXD, and in escape+, CPZ, CRTAP19-5, CFAP58." - should it be "A few genes were" ? In general, this sentence could benefit from a rewrite

Line 196: "...but not in KRAS respect to all mutations" - should this be KRAS, with respect to all mutations"? Also "Such result ..." should be either 'Such a result' or 'This result'

Line 197: "possibly forcing the accumulation of somatic mutations in hotspots that, when translated to protein, will be less

immunogenic." - It seems much more likely that hotspot mutations are driven by positive selection for gain of function than by purifying selection against other mutations. There is also evidence that some driver mutations are associated with immune evasion which could allow them to persist even if they were immunogenic. It's possible that some gof mutations are stronger than others and/or occur at sites that are not well presented. This could be assessed by looking at HLA binding affinity for peptides overlapping the different mutations if the authors want to argue this. Otherwise, the authors may wish to temper the speculation in this section, especially since they only focus on a couple genes.

Line 267 has a double space

Consider increase text size in figures in general. Fig 2 panels E and F could be made larger.

Although the authors did not generate new data or develop a completely new method, it would be nice to make the code supporting the analysis publicly available.
